# Customized flexible hollow microneedles for psoriasis treatment with reduced‐dose drug

**DOI:** 10.1002/btm2.10530

**Published:** 2023-05-02

**Authors:** Yingjie Ren, Junshi Li, Yiwen Chen, Jing Wang, Yuxuan Chen, Zhongyan Wang, Zhitong Zhang, Yufeng Chen, Xiaoyi Shi, Lu Cao, Jiayan Zhang, Huang Dong, Cong Yan, Zhihong Li

**Affiliations:** ^1^ National Key Laboratory of Science and Technology on Micro/Nano Fabrication School of Integrated Circuits Beijing China; ^2^ Beijing Advanced Innovation Center for Integrated Circuits Beijing China; ^3^ School of Life Sciences Beijing University of Chinese Medicine Beijing China; ^4^ College of Engineering Peking University Beijing China

**Keywords:** drug delivery, dual‐molding process, hollow microneedles

## Abstract

Microneedles, especially hollow microneedles (HMNs), play an important role in drug delivery, but most of the current HMNs are manufactured based on silicon microfabrication (lithography, etching, etc.), which are slightly conservative due to the lack of low‐cost, batch‐scale and customized preparation approach, especially for the HMNs with flexible substrate. For the first time, we propose the use of a high‐precision 3D printed master mold followed by a dual‐molding process for the preparation of HMNs with different shapes, heights, and inner and outer diameters to satisfy different drug delivery needs. The 3D printed master mold and negative mold can be reused, thereby significantly reducing the cost. HMNs are based on biocompatible materials, such as heat‐curing polymers or light‐curing resins. The thickness and rigidity/flexibility characteristics of the substrate can be customized for different applications. The drug delivery efficiency of the fabricated HMNs was verified by the in situ treatment of psoriasis on the backs of mice, which required only a 0.1‐fold oral dose to achieve similar efficacy, and the associated side effects and drug toxicity were reduced. Thus, this dual‐molding process can reinvigorate HMNs development.

## 1 INTRODUCTION

The application of microneedles, as an emerging drug delivery tool, has gained momentum in recent years.^1^, ^2^, ^3^, ^4^ The principle of microneedle drug delivery is that the microneedle tip punctures the stratum corneum of the skin to deliver a drug to the subcutaneous tissue to exert the effects of the drug.^5^, ^6^, ^7^, ^8^, ^9^ Typically, microneedles range in height from a few hundred microns to over a thousand microns, and because of their small height, they do not reach the capillaries and therefore do not cause bleeding and are virtually painless.^10^, ^11^, ^12^, ^13^, ^14^ This method has many advantages, such as reducing the use of traditional syringe needles, avoiding contamination and infection, allowing patients to self‐administer medications anywhere and anytime, reducing dependence on healthcare professionals and specific sites, and allowing large molecule drugs to pass through the stratum corneum, avoiding the low utilization of oral administration.^15^, ^16^, ^17^, ^18^, ^19^

There are three main classifications of microneedles: solid, dissolving and hollow microneedles.^20^, ^21^, ^22^ Solid microneedles are removed after piercing the skin, at which time some small holes will be created on the skin surface, and the drug is applied on top of the small holes. The drug will enter the subcutaneous tissue along the small holes to exert its medicinal effect, but the human skin has self‐repair abilities, and the small holes will close within a certain period of time, which will then prevent further drug diffusion, so solid microneedle drug delivery efficiency is low.^23^, ^24^, ^25^ Dissolving microneedles are made by mixing a drug with biodegradable polymer and curing so that the resulting microneedles exerts certain mechanical properties when piercing the skin. After piercing into the subcutaneous tissue, the tissue fluid dissolves the polymer, which in turn releases the drug. The needle tip is smaller in size and does not contain much of the drug, so its delivery efficiency is slightly improved.^26^, ^27^, ^28^, ^29^ HMNs can be understood as the miniaturization and arraying of traditional syringes and can be administered through an external syringe pump after piercing into the skin, and the microneedle drug delivery volume can theoretically reach the maximum tolerance of the skin, so the HMNs drug delivery efficiency and dose are higher compared to those of the first two, but their development and application are hindered because of the lack of a low‐cost, batch‐scale and customized manufacturing process.^30^, ^31^, ^32^, ^33^ Therefore, a new process is urgently needed to give new vitality to HMNs.

The main HMNs preparation processes are electroplating,^34^ laser drilling,^35^ deep reactive ion etching (DRIE),^36^ etching,^37^ evaporation^38^ and lithography,^39^ which are complex, time‐consuming and costly, and the main materials used are silicon^40^ and metals.^41^ The former is brittle and has a risk of fracturing in the skin, and the biocompatibility of both has yet to be verified for practical application.^42^, ^43^, ^44^, ^45^ In addition, the appeal process is mostly a standard process with strict requirements for relevant parameters. Microneedle tip morphology is strongly consistent across different needles, such as conical shapes with all the same parameters, which is rather daunting for certain applications that require different morphologies and heights and lack certain flexibility. Recently, high‐precision 3D printing technology has overcome these disadvantages, but its long printing time, high cost, and nonbiocompatibility make it difficult to be practically applied.^46^, ^47^, ^48^, ^49^ The dual‐molding process is common in the preparation of dissolving microneedles and solid microneedles due to its simple operation. This process is difficult to apply for HMNs because they are hollow inside. To ensure the permeability of HMNs, there must be a vertical column protruding from the grooves of the negative mold. However, this column is a great challenge for the dual‐molding process, and it is easy to break or bend the column during operation, so it is necessary to optimize the microneedle structure and negative mold selection.

**FIGURE 1:**
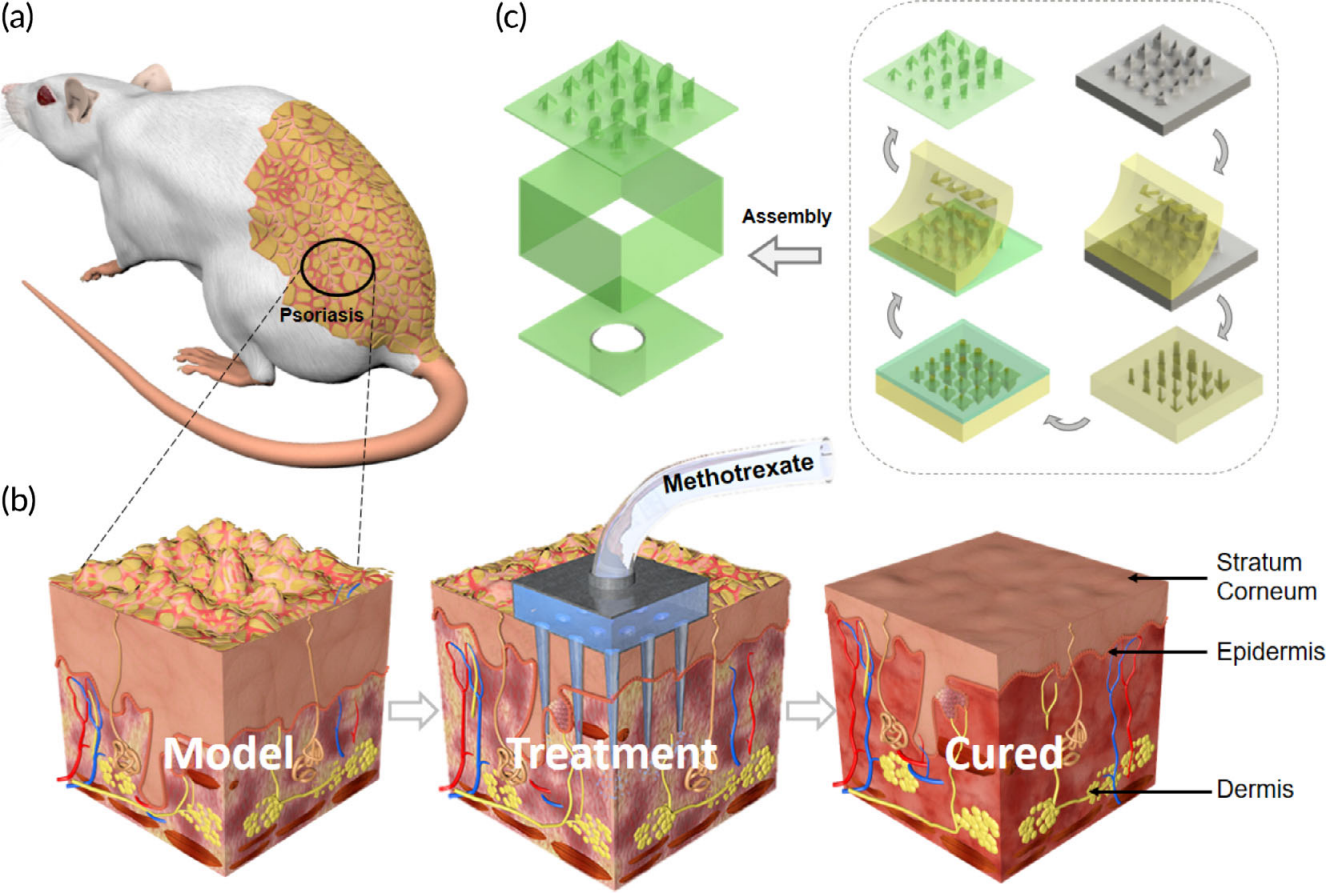
HMNs fabricated with a dual‐molding process and in vivo experiments. (a) Mouse with psoriasis on its back. (b) 3D structure of the skin during HMNs treatment. (c) HMN structural explosion and process flow diagrams.

For the first time, we propose a method using a dual‐molding process after high‐precision 3D printing, and this method combines the advantages of high‐precision 3D printing, and the time‐consuming problem is solved. We successfully prepared HMNs with different morphologies, heights and inner and outer diameters. The thickness and rigidity/flexibility characteristics of the substrate can be customized for different applications. When the 3D printed master mold and negative mold were prepared, the total time spent was just tens of minutes to hours depending on the material selected, significantly reducing the cost. To verify the usefulness of this method, psoriasis was generated on the backs of mice. The HMNs required only a 0.1‐fold oral dose to achieve similar efficacy, and the associated side effects and drug toxicity were reduced (Figure 1a,b). Therefore, HMNs based on this dual‐molding process have great application potential and research value.

## 2 RESULTS

### 2.1 HMNs prepared based on a dual‐molding process

The main HMNs structure consists of patches and microchambers (Figure 1c), both of which are adhered to the same material. The HMNs preparation process involves three components: a high‐precision 3D printed master mold, a negative mold with microcolumn arrays and grooves, and biocompatible HMNs. First, the HMNs were designed using Computer 3D software, and the files were exported to printing software, which sliced the files into layers every 10 μm in the Z‐direction and printed them layer by layer. The 3D printing equipment used for the 3D printed master mold was provided by Shenzhen MoFang Co., Ltd. The material used was high‐temperature resistant HTL yellowish low‐viscosity resin, which is sensitive to UV light at 405 nm. The length‐width‐height of the 3D printed master mold was 19 mm × 19 mm × 3.4 mm (3.2 mm), and 25 can be printed at once with a sample interval of 1 mm within 20–30 h. The precision of the equipment was 10 μm, so the layer‐by‐layer morphology of the needle tip surface appeared, which had no effect on the preparation process and practical applications. Fluoride solution was soaked overnight to ensure that any uncured resin remaining in the pinhole was cleaned, followed by heating at 50°C for 2 h to ensure its mechanical properties. The 3D printed master mold may have hindered resin curing,^50^ so a 2 μm thick film of Parylene C was deposited on the mold surface as a protective layer. A 3D printed master mold was placed into plastic petri dishes, and an Ecoflex/PDMS (E/P) mixture with a mass ratio of 10:1 was used as the negative mold material by comparing and calculating parameters, such as Young's modulus. The mold was vacuumed for 30 min until no bubbles emerged, and the liquid level was adjusted to approximately 5 mm above the needle tip and left for 10 min. The samples were heated at 80°C for 2 h to allow the E/P to finish curing. The blade was used to remove the additional E/P and then slowly uncovered along the four sides of the master mold to the edge of the array. Since the negative mold microcolumn array may break in the pinhole, both were immersed in sodium dodecyl sulfate solution (SDSS) for 10 min to reduce the adhesion between them, and slowly separated along the needle tip direction or vertically to form a negative mold with a microcolumn array and grooves. This step is repeated several times to obtain a large number of negative mold for batch manufacturing.

The negative mold was filled with liquid biocompatible materials until the liquid surface completely covered the microcolumn array and vacuumed for 10 min until no visible bubbles emerged. Residual bubbles were removed by a dropper, and then the liquid was slowly removed along the edge of the groove until the microcolumn array penetrated the liquid surface, the aspirated liquid can be reused and this step takes only tens of seconds to complete, and the remained liquid leave for 5 minutes to achieve natural leveling. Due to the surface tension, the liquid level was slightly higher at the edge of the groove and the microcolumn array, but this had no effect on the practical application. For the light‐curing polymer, transparent resin with a wavelength of 405 nm, low viscosity and high stiffness was selected and cured by UV light irradiation for 10 min, which was immersed in SDSS solution and uncovered following similar operations to form a light‐curing HMNs patch. The back interfaces were prepared in the same way, aligning and gluing them with the same resin, and thin tubes were used as channels to connect the HMNs to the syringe, forming HMNs on a rigid (resin) substrate. For the heat‐curing polymers, nonphotosensitive polyimide (PI) with a low viscosity and flexibility was selected, and the curing conditions were 140°C for 5 h followed by 240°C for 5 h. The operations were similar except for the curing conditions, allowing HMNs to form on a flexible (PI) substrate, which reduces the cost compared to the traditional process. Parameters such as HMNs shape, height, inner and outer diameter, thickness and the rigidity/flexibility characteristics of the substrate can be customized for different applications. This paper shows 2 types of HMNs, the first having a common shape reported in the literature with heights of 0.8, 1, 1.2, and 1.4 mm, bottom diameters and bottom edges of half the height, rounded (radius = 0.3 mm) at the root of each needle tip to enhance mechanical support. Four commonly used HMNs morphology parameters, square + cone, cylindrical + cone, rhombic and cylinder, were prepared. For square + cone and cylindrical + cone, as the height increases, their inner diameters are 0.2 mm, 0.2 mm, 0.2 mm, and 0.25 mm in order. For rhombic, as the height increases, their inner diameters are 0.25 mm, 0.3 mm, 0.35 mm, and 0.4 mm in order. For cylinder, as the height increases, their inner diameters are 0.35 mm, 0.4 mm, 0.45 mm, and 0.5 mm in order. The first two have a cone and the latter two appear sloped at half of the height to balance drug delivery volume and penetration force.

### 2.2 Characterization of HMNs fabricated with a dual‐molding process and biocompatible materials

**FIGURE 2:**
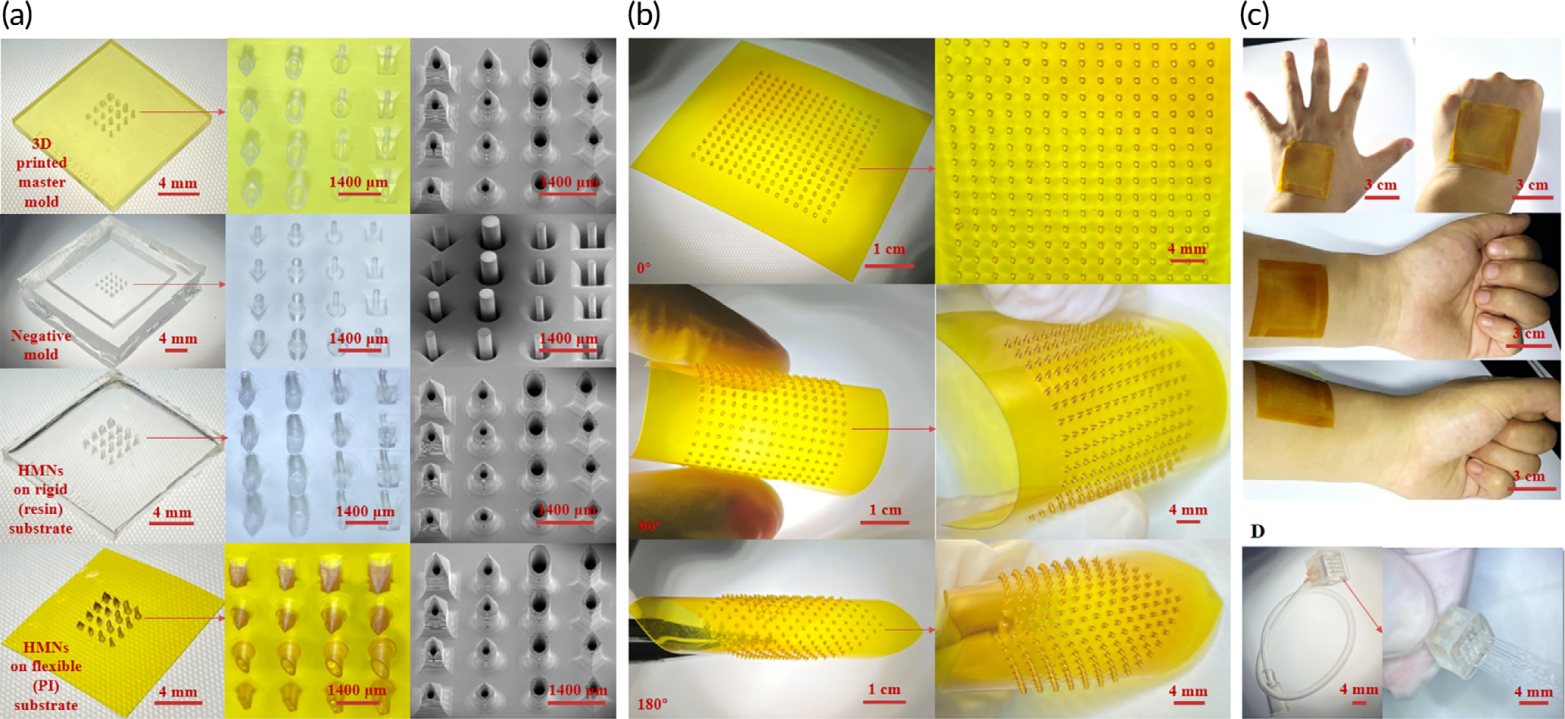
Characterization of the dual‐molding process and HMNs. (a) Characterization images of the 3D printed master mold, negative mold, resin‐HMNs, and PI‐HMNs. (b) Bending images of large‐area arrays of PI‐HMNs at different angles. (c) Characterization of large‐area arrays of PI‐HMNs on the back of the human hand and arm with different movements and sites to verify their great fit to the body surface. (d) Overall resin‐HMNs device diagram. Water flows uniformly from each pinhole to verify process stability.

**FIGURE 3:**
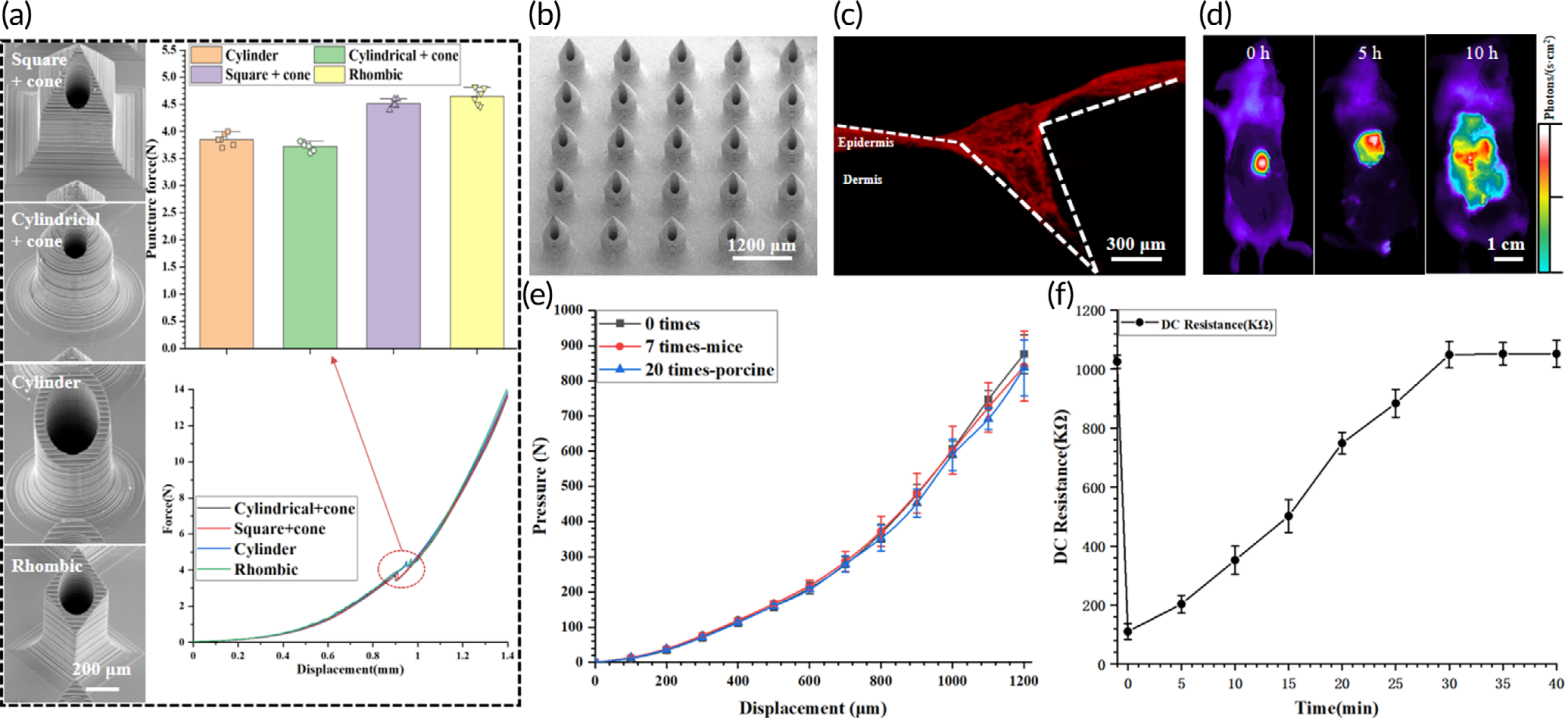
Practical use and characterization of resin‐based HMNs. (a) Ex vivo pig skin penetration experiments and penetration force of different morphologies. (b) Resin‐based HMNs of cylindrical + cone shape for experiments. (c) Ex vivo pig skin experiments verified the penetration ability of resin‐based HMNs. (d) Rhodamine B diffuses throughout the backs of mice within 10 h to verify the efficiency of the resin‐based HMNs. (e) The mechanical properties of the resin‐based HMNs are almost unchanged before and after use to verify the hardness of the material. (f) The pinhole in a human arm recovers within 30 min based on the recovery of the DC resistance.

Different needle tip shapes may be suitable for different scenarios, for example, square/cylindrical + cone has sharp tip with slightly smaller hole, which is less likely to be blocked after penetration and has less penetration force, and may be more suitable for surface drug delivery. Rhombic and cylinder has a gentle tip with a slightly larger hole and slightly more penetration force, and may be more suitable for implant drug delivery.^51^ Cylindrical and rhombic‐shaped HMNs have slightly large internal diameters, gentle tips, and large penetration forces that may be more suitable for implantable drug delivery applications. The tip size of the 3D printed master mold, which has a high flexibility, can be designed according to the desired application. The material of the negative mold was E/P, which is commonly used for the preparation of various molds and has a certain heat resistance. For the negative molds, the out‐of‐plane height of the microcolumn array was 0.6 mm, which can be flexibly changed according to requirements. Due to the uniqueness of the process, the 3D printed master mold and negative mold can be used repeatedly to meet low‐cost and batch requirements (Figure 2a). Cylinder PI‐based HMNs with an array size of 15 × 15, height of 1 mm, inner and outer diameters of 0.3/0.5 mm, and a circular center distance of 2 mm were prepared, and their flexibility was fully verified by bending at different angles (Figure 2b), the fit of the PI‐HMNs to the skin surface is demonstrated by different movements on the back of the hand and different positions on the arm (Figure 2c). The overall resin‐based HMNs device diagram and water flow test verify the degree of penetration of each pinhole and that water can flow uniformly out of each pinhole (Figure 2d), and its close‐up view of the tip of light‐curing resin HMNs (SI Appendix, Figure S1). Psoriasis causes the skin to thicken and harden, so the resin‐based HMNs were used for experiments. The needle length was 1.2 mm according to the characteristics of the onset of psoriasis in mice. Ex vivo pig skin penetration experiments were used to select HMNs with less penetration force, and four types of HMNs with a height of 1.2 mm could be successfully penetrated by mutation point (penetration force), because psoriasis causes the skin surface to harden and thicken, HMNs with less penetration force is required to ensure adequate penetration. The penetration force of square/cylindrical + cone is less than that of rhombic/cylinder because of its sharp tip and small penetration angle, and the penetration force of cylindrical + cone is slightly less than that of square + cone because the tip surface is probably smoother, but the difference is not significant. Psoriasis thickens the skin and makes it difficult for the needle tip to penetrate, so a shape with less penetration force should be chosen for the experiment (Figure 3a). Therefore, cylindrical + cone HMNs were prepared and used for all subsequent experiments (Figure 3b). The HMNs were used to perform pig skin piercing experiments by spraying rhodamine B (RB) on the needle tip surface. Due to the elasticity of the skin, the penetration depth was less than the needle tip length, which was approximately 1 mm (Figure 3c). The in vivo fluorescence imaging of mice verified the drug diffusion within the skin after HMNs administration. RB was used as the fluorescent dye, which was observed to cover essentially the entire back skin of the mice within 10 h (Figure 3d). Additionally, an Instron 5843 series single column mechanics tester was used to test the mechanical properties of the HMNs. The HMNs were placed horizontally on the sample table with the tips facing upward vertically, and the probe was pressed downward vertically at a speed of 120 μm/min. The mechanical range was 0–1000 N. The HMNs maintained their mechanical effect and shape after 7 and 20 repeated uses (Figure 3e). To verify the recovery of the needle holes after puncture, the arms of 3 volunteers were used as tests. Two identical HMNs were simultaneously used to puncture the skin at 1 cm intervals, the DC resistance was tested using a gel wet electrode on the resulting needle holes, and the resistance values recovered within 30 min to demonstrate the recovery of the skin (Figure 3f). To accommodate the size of the cell culture plate, a circular sheet‐shaped light‐cured resin with diameter of 5 mm and thickness of 1 mm was completely submerged in the cell solution and cell activity was observed at 12 h, 24 h, 36 h, and 48 h, respectively. NIH3T3 cells were used to verify the biosafety of the HMNs, and the results showed that the light‐cured resin is highly biocompatible (SI Appendix, Figure S2). The 3D printed master mold and negative mold showed almost no change in morphology after 20 uses and could continue to be used, further verifying the maturity of the process. After the ex vivo pig skin and in vivo mice experiments, HMNs were not broken or bent, fully demonstrating their utility and safety (SI Appendix, Figure S3). The in vivo experiments showed that HMNs require only a 0.1‐fold oral dose to achieve similar therapeutic effects, much less than the 0.5‐fold dose of dissolving microneedles.^52^ After the preparation process and in vivo experiments, it was fully verified that the HMNs based on the dual‐molding process have great application prospects and application value.

### 2.3 In vivo experiments and characterization

**FIGURE 4:**
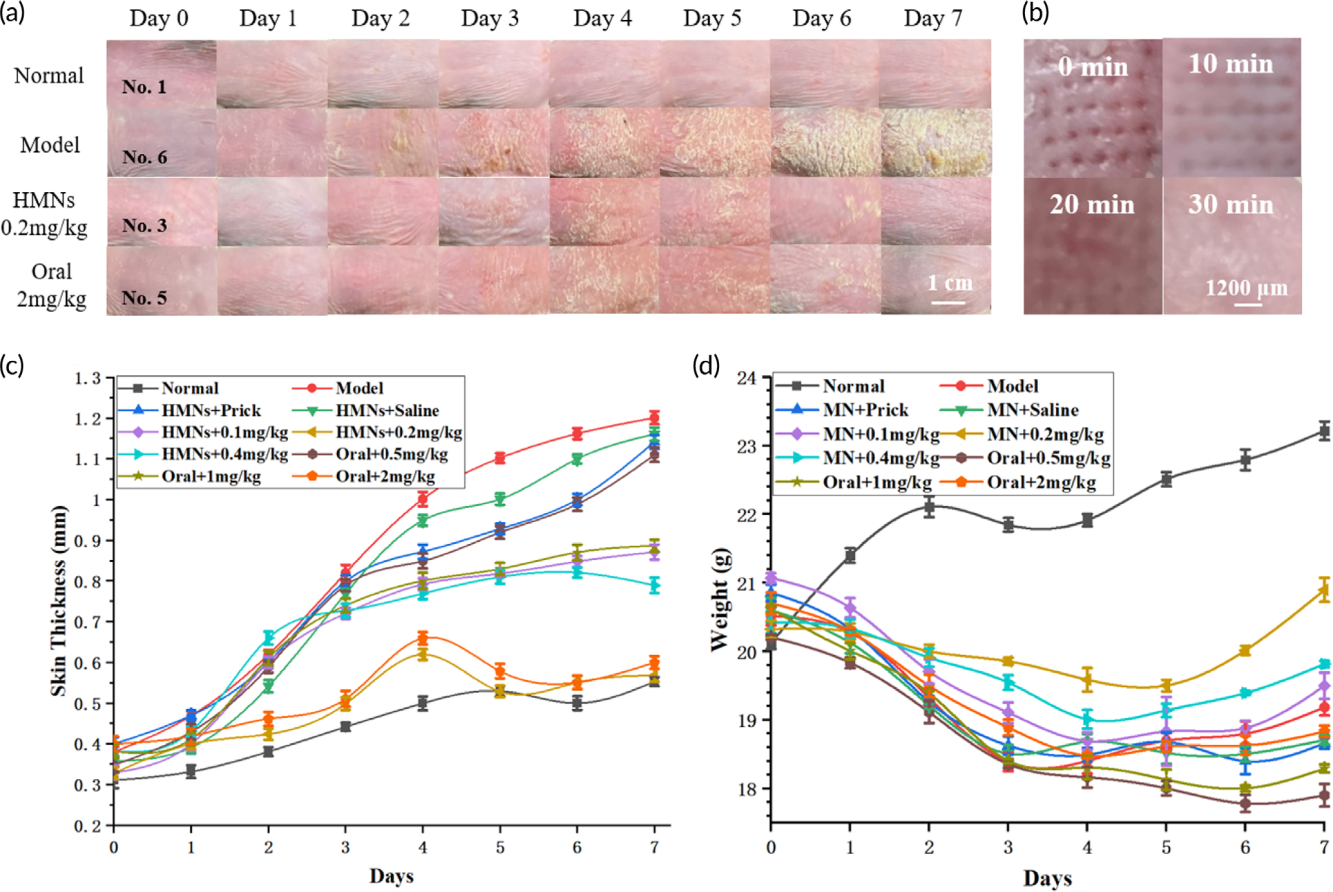
Recovery of the backs of mice. (a) Visualization of part of the dorsal skin of mice. (b) Pinhole recovery within 30 min on mouse skin. (c) Changes in bilayer thickness quantitatively reflect the degree of skin recovery. HMNs +0.2 mg/kg and oral +2 mg/kg showed similar changes and had the best therapeutic effect. (d) Weight change from 0–7 days. HMNs +0.2 mg/kg was the experimental group with the best weight recovery.

Five‐week‐old male BALB/C mice weighing 20 g were used for in vivo experiments and were divided into 10 groups of 6: normal, model, HMNs + prick only, HMNs + saline, HMNs + methotrexate (MTX) 0.1 mg/kg (dose/weight), HMNs + MTX 0.2 mg/kg, HMNs + MTX 0.4 mg/kg, oral + MTX 0.5 mg/kg, oral + MTX 1 mg/kg and oral + MTX 2 mg/kg. The modeling approach, oral dose grouping and HMNs dose grouping were referenced from the literature.^52^ The backs of the mice were shaved 3 days before the experiment, and an area larger than or equal to 2 cm × 2 cm was left bare. The modeling was started at 18:00 each day on days 0–6, and the normal group was evenly coated with Vaseline over an area the size of a soybean pellet, while the model and experimental groups were evenly coated with the same amount of 5% imiquimod for the induction of psoriasis. Treatment was conducted daily on days 1–7 at 8:00. The HMNs group was administered 20 μL at one‐time injection at one site, and the oral group was administered 100 μL using gavage. HMNs are disinfected after each use according to the manufacturer's recommendations and actual needs (75% alcohol soak + UV disinfection, respectively 5 min), which will not damage the device. Sampling and analysis was conducted from 8:00 to 15:00 on day 8. Daily changes in the skin appearance, pinhole recovery, bilayer skin thickness, body weight, splenic index, Psoriasis area and severity index (PASI) and related physiological analysis parameters of mice for statistical analysis. Skin appearance changes and bilayer skin changes (Figure 4a,c and SI Appendix, Figure S4) were used to demonstrate the efficacy of HMNs and oral administration. The skin of the normal group remained consistent every day, and the bilayer skin thickness increased slowly. In the model group, the skin became redder and thicker every day until large silver flakes grew. The HMNs + prick only group and HMNs + saline group were used as the reference group and exhibited almost no therapeutic effect on the disease, and their skin and bilayer skin thickness changes were almost equal to those of the model group. In the HMNs + MTX 0.1, 0.2, and 0.4 mg/kg groups, it can be seen that although 0.1 mg/kg had a certain therapeutic effect, it did not have a good therapeutic effect because of the small drug amount. HMNs +0.2 mg/kg had the best therapeutic effect because of the moderate drug amount. HMNs +0.4 mg/kg is less effective than 0.2 mg/kg, possibly because the drug is already saturated and excess drug may not be effective and remain in the subcutaneous tissue, which in turn may cause further skin damage. The damaged skin was more likely to induce psoriasis after modeling the same night, resulting in slightly poorer efficacy. In the oral +0.5, 1, and 2 mg/kg MTX groups, the efficacy was better with increasing dosage. Because a portion of the drug will be degraded by the gastrointestinal tract after oral administration, the remaining portion will be absorbed into the body and transported throughout the whole body in circulation, and only a small portion of the drug will arrive at the onset site, so an efficacy similar to that of HMNs +0.2 mg/kg was achieved by oral +2 mg/kg. In addition, assuming a psoriasis onset area of 4 cm^2^, the HMNs required only 1 μg/cm^2^ MTX to achieve a good therapeutic effect, much lower than the approximately 3.5 μg/cm^2^ dissolving microneedles,^52^ and the drug delivery efficiency was greatly enhanced. Needle hole recovery (Figure 4b) was seen to be complete within 30 min after removal of the HMNs. Body weight changes (Figure 4d) also indirectly reflected the degree of health. The normal group had a slow increase in body weight almost every day, reflecting their highest level of health. Their HMNs administration was lower, and drug toxicity had less of an effect on body weight, which decreased and then increased as the disease was cured. The oral group was administered a large drug amount directly to the stomach, which had a great impact on the living condition of the mice, so their body weight decreased quickly, and their health was poor. The model and reference groups exhibited almost no therapeutic effect, and their body weight changes and degree of health were slightly worse than those of the oral administration groups.

**FIGURE 5:**
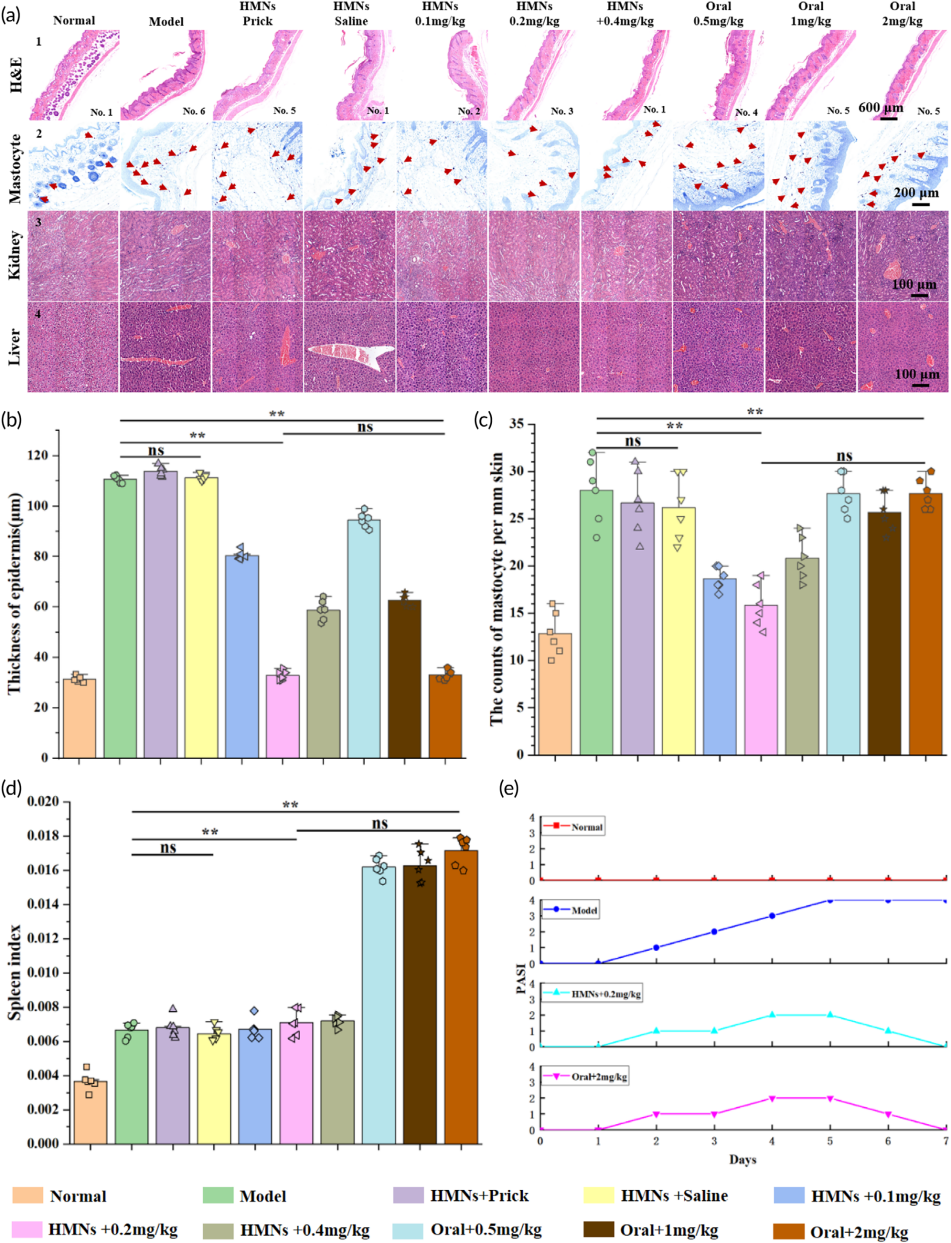
Pathological analysis of in vivo experiments. (a) Analysis of H&E, mastocyte counting, kidney and liver. (b) Epidermis thickness analysis. (c) Mastocyte counting. (d) Splenic index. (e) PASI score (*n* = 6, mean ± SDs) (*ns*, “*,” “**,” and “***” indicate no significance, *p* < 0.05, *p* < 0.01, and *p* < 0.001).

H&E staining, mastocyte counting, and kidney and liver sampling were performed in mice after euthanasia. H&E (Figure 5a_1_,b) was used to measure the epidermal thickness, and it was seen that HMNs + 0.2 mg/kg and oral + 2 mg/kg had similar results to the normal group. Mastocyte counting (Figure 5a_2_,c) was used to indicate skin allergies, and HMNs + 0.2 mg/kg had similar results to the normal group, indicating less allergy. The kidney and liver results (Figure 5a_3_,a_4_) showed that there was an increase in the number of blood vessels in all groups except the normal and HMNs + 0.1, 0.2, and 0.4 mg/kg groups, which also indirectly indicated great damage to the liver and kidney. The spleen index (Figure 5d) results showed large values in the oral group, indicating greater damage to the body. The PASI score (Figure 5e and SI Appendix, Table S1), which needed to be rated by some uninvolved person, was rated according to the visual changes of the skin, and the integer score of healthy to the most severe condition was from 0 to 4. This paper used three uninvolved persons for scoring, and their results were consistent. Relevant physiological analyses demonstrated the good efficacy of small HMNs doses, which also had fewer side effects, such as biological toxicity. Serum results (SI Appendix, Figure S5) were used as a quantitative analysis of liver and kidney function, and it can be seen that in terms of ALT, AST, BUN, CREA and O/P, HMNs+0.2 mg/kg always remained almost the same as the normal group, i.e., there was less of an impact on the liver and kidney function and good health. The kidney diagram (SI Appendix, Figure S6) shows the surface whitening in the model, reference and oral groups, but that of the oral group was the most obvious, which also indicated large amounts of body damage. The in vivo experiments fully validated the superior drug delivery ability of HMNs.

## 3 DISCUSSION

With the rise in microneedle drug delivery, the microneedle preparation process has gradually become important. Compared with the preparation processes of solid microneedles and dissolving microneedles fabricated with a dual‐molding process, the preparation process of HMNs has disadvantages of a higher production cost and time consumption and low flexibility because of processes including lithography, etching, DRIE and complex plating, which makes it difficult to realize low‐cost and batch‐scale production. Among the categories of microneedle drug delivery that have been industrialized, dissolving microneedle drug delivery has dominated due to the simple preparation process of dissolving microneedles.

For the first time, we prepared HMNs with different morphologies, heights, inner and outer diameters, substrate thicknesses and rigidity/flexibility values using a dual‐molding process, which greatly enhances HMNs flexibility. The 3D printed master mold and negative mold are reusable, and the whole preparation process takes only tens of minutes to hours depending on the material selection with a well‐prepared 3D printed master mold and negative mold, which greatly reduces the cost and time and has great application prospects.

The HMNs were made with biocompatible materials, such as light‐curing and heat‐curing materials, and the thickness and rigidity/flexibility characteristics of the substrate can be customized for different applications. PI was chosen as the heat‐curing flexible material, whose flexibility can be verified by bending and subsequent recovery at different angles. Resin was chosen as the light‐curing rigid material. The above two materials were used to validate the unique advantages of the process. Psoriasis hardens and thickens the skin, so a light‐curing rigid material was used for the experiments. This type of material was originally used for dental repair and has a high hardness that can easily pierce pig skin. To verify the recovery of the pinholes after puncture, the arms of three volunteers were used as experimental subjects, and the recovery of the pinhole within 30 min was observed by the change in DC resistance. In vivo fluorescence imaging verified that the drug could diffuse throughout the backs of mice within 10 h, demonstrating extremely high drug utilization. Water flow uniformly and consistently exited from each pinhole, verifying the stability of the process. Mechanical testing quantitatively verified that the HMNs were undamaged before and after use. HMNs based on the dual‐molding process have the advantage of low cost and batch size, and can be prepared in different morphologies and array sizes depending on the site of drug delivery, and materials can be selected according to different degrees of damaged skin surfaces. The feasibility of the process was demonstrated by the use of HMNs based on light‐curing resin for the treatment of psoriasis in mice, which has a large area of onset and causes the skin to thicken and harden until silver flakes appear. Psoriasis was generated on the backs of mice, and a treatment drug was administered in situ using HMNs for a 7‐day experimental period, with treatment in the morning and modeling in the evening each day until day 8. Ten groups were used for the experiment after taking into account the effects of all variables. The efficacy of HMNs + MTX 0.2 mg/kg and oral + 2 mg/kg was similar and assessed by daily visual skin recovery, bilayer skin thickness measurements, body weight changes, PASI scores and so on, and the mice in the HMNs group had a higher body weight and better health than the mice in the oral group.

In conclusion, we realized for the first time HMNs preparation by a dual‐molding process and verified the advantages and stability of the process by experiments such as pig skin piercing, DC impedance change, in vivo fluorescence imaging, water flow and mechanical curve analysis. Through the treatment of psoriasis, it was verified that the efficacy of HMNs can be equal to or even greater than the efficacy of mainstream microneedles. Therefore, HMNs are expected to be applied in more practical applications.

## 4 MATERIALS AND METHODS

### 4.1 Materials

High‐precision 3D printing resin (Yellow‐20) was purchased from Shenzhen Mofang New Material Technology Company. Silicone elastomer PDMS (SYLGARD™ 184) was purchased from Dow Chemical Company. Ecoflex 00‐30 was purchased from SMOOTH‐ON. Light‐curing biocompatible resin (iF 3162) was purchased from Guangzhou Ifun Technology Company.

### 4.2 High‐precision 3D printing and sample postprocessing

The design files were modeled in 3D using a computer and then exported as STL files with an accuracy of 10 μm. Magics Print for BMF 23.2 was used to slice the files layer by layer at 10 μm in the *Z*‐direction. The resulting samples were removed and soaked with fluoride solution overnight (10 h), blown dry and heated in an oven at 50°C for 2 h to completely cure and enhance the mechanical properties. PDS 2035 equipment was used for the vapor deposition of Parylene C films. Five grams of raw material corresponded to approximately 1 μm thickness, and we used 10 g (2 μm) to completely isolate and protect the sample.

### 4.3 Fabrication of HMNs systems

After the HMNs patch and the back connection structure were prepared, the two were aligned, and a layer of biocompatible resin was applied at the junction as glue. The two were placed under a UV lamp of 405 nm for 5 min to cure, and the excess was cut off with scissors and polished slowly with sandpaper. A TYD02‐02 syringe pump was purchased from Baoding LEADFLUID Technology Company, and the speed was set to 5 μL/min. An infusion tube was attached to the HMNs system in the same way and used to connect the syringe pump. The total elapsed time for single mice was less than 5 min, with good efficiency.

### 4.4 In vivo experimental details

Mice were divided into 10 groups with 6 mice per group and managed according to the animal management method of Beijing University of Chinese Medicine, with bedding and food changed daily and ventilation maintained at all times. The hair removal cream Silk & Fresh was purchased from Veet, France, and used for hair removal on the backs of the mice. Sodium pentobarbital (0.3%) was used as an anesthetic, and approximately 100 μL was injected at a time according to body weight. A 5% imiquimod cream was purchased from 3 M Health Care Limited, UK, and used for modeling psoriasis. Methotrexate was purchased from Shanghai ShangPharma Xinyi Pharmaceutical Company for the treatment of psoriasis and was dosed with saline at different concentrations to delineate different experimental groups. Alcohol (75%) and UV disinfection was used to disinfect the environment and HMNs system. Oral administration was performed by professional staff through a gavage needle. The HMNs group was administered by pressing for 1 min after pricking and 2 min of administration time and then pressing for 1 min. Photographs were taken from above by an iPhone 13 (black, 256 G) at a height of 15 cm.

### 4.5 Recording and analyzing in vivo experiments

Vernier calipers were used to perform daily double skin thickness measurements on the dorsal surfaces of the mice, and the average was taken after measuring three different locations. An in vivo fluorescence imaging system comprising PerkinElmer's IVIS Lumina SRMS Series product line was used to observe the subcutaneous distribution of rhodamine B in mice. The mice were subjected to an orbital extraction of 1 mL of blood into anticoagulation tubes after removal of the right eyeball. The blood samples were centrifuged at 4°C and 3000 rpm, and serum (300 μL) was separated at high speed for 15 min before liver and kidney function indexes were measured. After blood collection, the mice were fixed in the supine position on a mouse plate, the thorax was quickly opened, excess connective tissue was separated and removed, the spleen was dissected, and the residual blood was gently aspirated from the surface with filter paper. The mice were weighed and photographed and then stored in a −80°C refrigerator. The redundant connective tissues of the mice were separated and removed, the liver was dissected, the residual blood was gently aspirated from the surface with filter paper, the largest lobe of the liver was fixed with 4% paraformaldehyde (PA) to prepare pathological sections, and the rest of the liver tissues were stored at −80°C in the refrigerator. The right kidney was dissected, and the residual blood was gently aspirated from the surface with filter paper. The kidney was cut crosswise, half of the kidney tissue was fixed with 4% PA, and pathological sections were prepared. The whole layer of skin on the dorsum of mice (2 cm × 2 cm) was cut off and fixed with 4% PA to prepare pathological sections. The mouse spleen organs were removed intact and weighed on an electronic balance, and the spleen organ coefficients were calculated according to the formula: spleen organ coefficient = spleen mass (g)/body mass (kg). Pathological tissue changes: liver, kidney, skin behind the ear, and ear tissues were taken and fixed with 4% PA and then made into paraffin sections after dehydration, wax immersion, embedding, and the morphological changes of the tissues were observed by H&E staining. H&E staining was performed by taking paraffin sections of tissues and sequentially performing dewaxing, debenzene, hydration, hematoxylin staining, water washing, and color separation. After the sections appeared pink, were water washed and dyed, the sections were stained with ethanol and dehydrated from low to high concentration, after which the xylene was transparent, and finally the sections were sealed with neutral gum. Pathological sections were observed by randomly selecting five microscopic fields and photographing them at 4× and 10× magnification by technicians who were unaware of the experimental grouping to determine the damage to the liver and kidney tissues and the degree of skin keratinization and inflammatory cell infiltration. The PASI score is an important index for evaluating the severity of psoriasis in clinical and pharmacological studies and includes four aspects: erythema, scaling, infiltration and total score (i.e., erythema score + scaling score + infiltration score). Erythema, scaling and infiltration were scored as 0, 1, 2, 3, and 4, respectively, according to the severity of psoriasis development, which corresponded to the symptomatology of none, mild, moderate, severe and extremely severe. This scoring was evaluated by three students with different specialties, and the mean value of each index was obtained from the results. A trend line was drawn, thus monitoring the severity of psoriasis in each group of mice.

### 4.6 Statistical analysis

Differences between two independent groups were calculated using *t* test. *p* values less than 0.05 were considered statistically significant and are denoted as follows: *ns*, no significance, *p < 0.05, **p < 0.01, and ***p < 0.001. All data are plotted as the means ± SDs. All statistical analyses were performed using Origin 2020b (Origin Lab Corporation).

## AUTHOR CONTRIBUTIONS

**Yingjie Ren:** Conceptualization (equal); data curation (lead); formal analysis (lead); investigation (lead); methodology (lead); project administration (lead); resources (lead); software (lead); validation (lead); visualization (lead); writing – original draft (lead). **Junshi Li:** Investigation (supporting). **Yiwen Chen:** Investigation (supporting). **Jing Wang:** Investigation (supporting). **Yuxuan Chen:** Investigation (supporting). **Zhongyan Wang:** Investigation (supporting). **Zhitong Zhang:** Investigation (supporting). **Yufeng Chen:** Investigation (supporting). **Xiaoyi Shi:** Investigation (supporting). **Lu Cao:** Investigation (supporting). **Jiayan Zhang:** Investigation (supporting). **Huang Dong:** Conceptualization (supporting); investigation (supporting). **Cong Yan:** Investigation (supporting). **Zhihong Li:** Conceptualization (lead); funding acquisition (lead); supervision (lead); writing – review and editing (lead).

## CONFLICT OF INTEREST STATEMENT

The authors have no conflicts of interest to declare.

### PEER REVIEW

The peer review history for this article is available at https://www.webofscience.com/api/gateway/wos/peer-review/10.1002/btm2.10530.

## Supporting information


**Data S1.** Supporting Information

## Data Availability

The authors declare that all data supporting the findings of this study are available within the paper. Other raw and analyzed data generated during the study are available from the corresponding authors on reasonable request for research purposes.

## References

[1] Wang ZJ , Wang JQ . Dual self‐regulated delivery of insulin and glucagon by a hybrid patch. PNAS. 2020;44:29512‐29517. 10.1073/pnas.2011099117 PMC7703584 33177238

[2] Zhu JX , Zhou XW . Gelatin methacryloyl microneedle patches for minimally invasive extraction of skin interstitial fluid. Small. 2020;16:1905910. 10.1002/smll.201905910 PMC7182487 32101371

[3] Yang G , Chen Q . A therapeutic microneedle patch made from hair‐derived keratin for promoting hair regrowth. ACS Nano. 2019;4:4354‐4360. 10.1021/acsnano.8b09573 30942567

[4] Makvandi P , Jamaledin R . Stimuli‐responsive transdermal microneedle patches. Mater Today. 2021;47:206‐222. 10.1016/j.mattod.2021.03.012 PMC9635273 36338772

[5] Li W , Terry RN . Rapidly separable microneedle patch for the sustained release of a contraceptive. Nat Biomed Eng. 2019;3:220‐229. 30948808 10.1038/s41551-018-0337-4

[6] Tran KTM , Gavitt TD . Transdermal microneedles for the programmable burst release of multiple vaccine payloads. Nat Biomed Eng. 2021;5:998‐1007. 33230304 10.1038/s41551-020-00650-4

[7] Sheng T , Luo BW . Microneedle‐mediated vaccination: innovation and translation. Adv Drug Deliv Rev. 2022;179:113919. 10.1016/j.addr.2021.113919 34375682

[8] Chi JJ , Sun LY . Chinese herb microneedle patch for wound healing. Bioactive Mater. 2021;6:3507‐3514. 10.1016/j.bioactmat.2021.03.023 PMC7988348 33817424

[9] Gao BB , Guo MZ . Intelligent silk fibroin based microneedle dressing (i‐SMD). Adv Funct Mater. 2020;31:2006839.

[10] Chen W , Wainer J . Dynamic omnidirectional adhesive microneedle system for oral macromolecular drug delivery. Sci Adv. 2022;8:eabk1792. 34985942 10.1126/sciadv.abk1792 PMC8730401

[11] Ester C‐S , Kim S . A microneedle platform for buccal macromolecule delivery. Sci Adv. 2021;4:eabe2620. 10.1126/sciadv.abe2620 PMC10964974 33523951

[12] Lin L , Wang YQ . Multimicrochannel microneedle microporation platform for enhanced intracellular drug delivery. Adv Funct Mater. 2021;32:2109187.

[13] Tang JN , Wang JQ . Cardiac cell‐integrated microneedle patch for treating myocardial infarction. Sci Adv. 2018;4:eaat9365. 30498778 10.1126/sciadv.aat9365 PMC6261659

[14] Guo MZ , Wang YQ . Shark tooth‐inspired microneedle dressing for intelligent wound management. ACS Nano. 2021;15:15316‐15327. 34533924 10.1021/acsnano.1c06279

[15] Chen W , Cai B . Reducing false negatives in COVID‐19 testing by using microneedle‐based oropharyngeal swabs. Matter. 2020;5:1589‐1600. 10.1016/j.matt.2020.09.021 PMC7535620 33043290

[16] Wan T , Pan Q . Microneedle‐assisted genome editing: a transdermal strategy of targeting NLRP3 by CRISPR‐Cas9 for synergistic therapy of inflammatory skin disorders. Sci Adv. 2021;7:eabe2888. 33692106 10.1126/sciadv.abe2888 PMC7946375

[17] DeMuth PC , Min Y . Polymer multilayer tattooing for enhanced DNA vaccination. Nat Mater. 2013;12:367‐376. 23353628 10.1038/nmat3550 PMC3965298

[18] Wang JQ , Ye YQ . Core‐shell microneedle gel for self‐regulated insulin delivery. ACS Nano. 2018;12:2466‐2473. 29455516 10.1021/acsnano.7b08152 PMC6037424

[19] Shi H , Zhou JH . A rapid corneal healing microneedle for efficient ocular drug delivery. Small. 2022;18:2104657. 10.1002/smll.202104657 35083856

[20] Rezvan J , Yiu CKY . Advances in antimicrobial microneedle patches for combating infections. Adv Mater. 2020;32:e2002129. 32602146 10.1002/adma.202002129

[21] Xuan J , Zhu DD . Insulin delivery systems combined with microneedle technology. Adv Drug Deliv Rev. 2018;127:119‐137. 29604374 10.1016/j.addr.2018.03.011

[22] Paredes AJ , Ramoller IK . Microarray patches: breaking down the barriers to contraceptive care and HIV prevention for women across the globe. Adv Drug Deliv Rev. 2021;173:331‐348. 33831475 10.1016/j.addr.2021.04.002

[23] Abbie O , Maryse B . Assessment of solid microneedle rollers to enhance transmembrane delivery of doxycycline and inhibition of MMP activity. Drug Deliv. 2017;1:942‐951. 10.1080/10717544.2017.1337826 PMC8241162 28618841

[24] Li QY , Zhang JN . A solid polymer microneedle patch pretreatment enhances the permeation of drug molecules into the skin. RSC Adv. 2017;25:15408‐15415.

[25] Madeleine W , Katja O . Feasibility study for intraepidermal delivery of proteins using a solid microneedle array. Int J Pharm. 2015;2:52‐58. 10.1016/j.ijpharm.2015.03.046 25819344

[26] Jungyoon O , Jang M . Dissolving candlelit microneedle for chronic inflammatory skin diseases. Adv Sci. 2021;8:2004873. 10.1002/advs.202004873 PMC8292898 34306973

[27] Balmert SC , Carey CD . Dissolving undercut microneedle arrays for multicomponent cutaneous vaccination. J Control Release. 2020;317:336‐346. 31756393 10.1016/j.jconrel.2019.11.023 PMC8237702

[28] Jang MY , Kang BM . High‐dose steroid dissolving microneedle for relieving atopic dermatitis. Adv Healthc Mater. 2021;10:2001691. 10.1002/adhm.202001691 33586358

[29] Long LY , Liu WQ . Dissolving microneedle‐encapsulated drug‐loaded nanoparticles and recombinant humanized collagen type III for the treatment of chronic wound via anti‐inflammation and enhanced cell proliferation and angiogenesis. Nanoscale. 2022;4:1285‐1295. 10.1039/d1nr07708b 35006234

[30] Sachan R , Schurch P . Hollow copper microneedle made by local electrodeposition‐based additive manufacturing. MRS Adv. 2021;6:893‐896.

[31] Yeung C , Chen S . A 3D‐printed microfluidic‐enabled hollow microneedle architecture for transdermal drug delivery. Biomicrofluidics. 2020;13:064125. 10.1063/1.5127778 PMC6906119 31832123

[32] Mathew E , Pitzanti G . Optimization of printing parameters for digital light processing 3D printing of hollow microneedle arrays. Pharmaceutics. 2022;13:1837. 10.3390/pharmaceutics13111837 PMC8622592 34834250

[33] Economidou SN , Uddin MJ . A novel 3D printed hollow microneedle microelectromechanical system for controlled, personalized transdermal drug delivery. Addit Manuf. 2021;38:101815.

[34] Miller PR , Moorman M . Fabrication of hollow metal microneedle arrays using a molding and electroplating method. MRS Adv. 2019;4:1417‐1426.

[35] Suzuki M , Takahashi T . 3D laser lithographic fabrication of hollow microneedle mimicking mosquitos and its characterization. Int J Nanotechnol. 2018;15:157‐173.

[36] Li Y , Zhang H . Fabrication of sharp silicon hollow microneedles by deep‐reactive ion etching towards minimally invasive diagnostics. Microsyst Nanoeng. 2019;5:41. 31636931 10.1038/s41378-019-0077-y PMC6799813

[37] Bolton CJW , Howells O . Hollow silicon microneedle fabrication using advanced plasma etch technologies for applications in transdermal drug delivery. Lab Chip. 2020;20:2788‐2795. 32632424 10.1039/d0lc00567c

[38] Trols A , Hintermuller MA . Drug dosage for microneedle‐based transdermal drug delivery systems utilizing evaporation‐induced droplet transport. Microfluidics Nanofluidics. 2019;23:91.

[39] Li R , Liu X . Fast customization of hollow microneedle patches for insulin delivery. Int J Bioprint. 2022;8:124‐135. 10.18063/ijb.v8i2.553 PMC9159536 35669318

[40] Resnik D , Mozek M . In vivo experimental study of noninvasive insulin microinjection through hollow Si microneedle array. Micromachines. 2018;9:40. 30393315 10.3390/mi9010040 PMC6187700

[41] Chen JM , Cheng P . A minimally invasive hollow microneedle with a cladding structure: ultra‐thin but strong, batch manufacturable. IEEE Trans Biomed Eng. 2020;66:3480‐3485. 10.1109/TBME.2019.2906571 30932818

[42] Hu ZL , Meduri CS . Solid and hollow metallic glass microneedles for transdermal drug‐delivery. Appl Phys Lett. 2020;116:203703.

[43] Zhang YH , Campbell SA . Finite element analysis of hollow out‐of‐plane HfO_2_ microneedles for transdermal drug delivery applications. Biomed Microdevices. 2018;20:19. 29455257 10.1007/s10544-018-0262-z

[44] Kim H , Theogarajan LS . A repeatable and scalable fabrication method for sharp, hollow silicon microneedles. J Micromech Microeng. 2018;28:035007.

[45] Mishra R , Maiti TK . Development of SU‐8 hollow microneedles on a silicon substrate with microfluidic interconnects for transdermal drug delivery. J Micromech Microeng. 2018;28:105017.

[46] Xenikakis I , Tsongas K . Fabrication of hollow microneedles using liquid crystal display (LCD) vat polymerization 3D printing technology for transdermal macromolecular delivery. Int J Pharm. 2021;597:120303. 33540009 10.1016/j.ijpharm.2021.120303

[47] Chantell F , Roman L . Three‐dimensional (3D) printed microneedles for microencapsulated cell extrusion. Bioengineering‐Basel. 2018;5:59. 30065227 10.3390/bioengineering5030059 PMC6164407

[48] Yadav V , Sharma PK . 3D printed hollow microneedles array using stereolithography for efficient transdermal delivery of rifampicin. Int J Pharm. 2021;605:120815. 34153441 10.1016/j.ijpharm.2021.120815

[49] Szeto B , Aksit A . Novel 3D‐printed hollow microneedles facilitate safe, reliable, and informative sampling of perilymph from Guinea pigs. Hear Res. 2021;400:108141. 33307286 10.1016/j.heares.2020.108141 PMC8656365

[50] Venzac B , Deng S . Why are 3D‐printed molds inhibiting PDMS curing. μTAS. 2020;2:282‐283.

[51] Ebrahiminejad V , Prewett PD . Microneedle arrays for drug delivery and diagnostics: toward an optimized design, reliable insertion, and penetration. Adv Mater Interfaces. 2022;9:2101856.

[52] Du HY , Liu P . Hyaluronic acid‐based dissolving microneedle patch loaded with methotrexate for improved treatment of psoriasis. ACS Appl Mater Interfaces. 2019;11:43588‐43598. 31651148 10.1021/acsami.9b15668

