# Supplementary material for: Customized flexible hollow microneedles for psoriasis treatment with reduced‐dose drug
Source: Bioeng Transl Med. 2023 May 2;8(4):e10530. doi: 10.1002/btm2.10530 (PMC10354769; doi:10.1002/btm2.10530)
Supplement: Supplementary file 1 — Data S1. Supporting Information [file BTM2-8-e10530-s001.docx]

**Supporting Information for**

Customized flexible hollow microneedles for psoriasis treatment with reduced-dose drug

Yingjie Ren^1,2^, Junshi Li^1,2^, Yiwen Chen^3^, Jing Wang^3^, Yuxuan Chen^1,2^, Zhongyan Wang^1,2^, Zhitong Zhang^1,2^, Yufeng Chen^1,2^, Xiaoyi Shi^1,2^, Lu Cao^1,4^, Jiayan Zhang^1,2^, Dong Huang^1,2^, Cong Yan^3^, Zhihong Li^1,2*^

Corresponding author: Zhihong Li, **Email:** [zhhli@pku.edu.cn](mailto:zhhli@pku.edu.cn)

**This PDF file includes:**

Supporting text

Figures S1 to S5

Tables S1 to S1


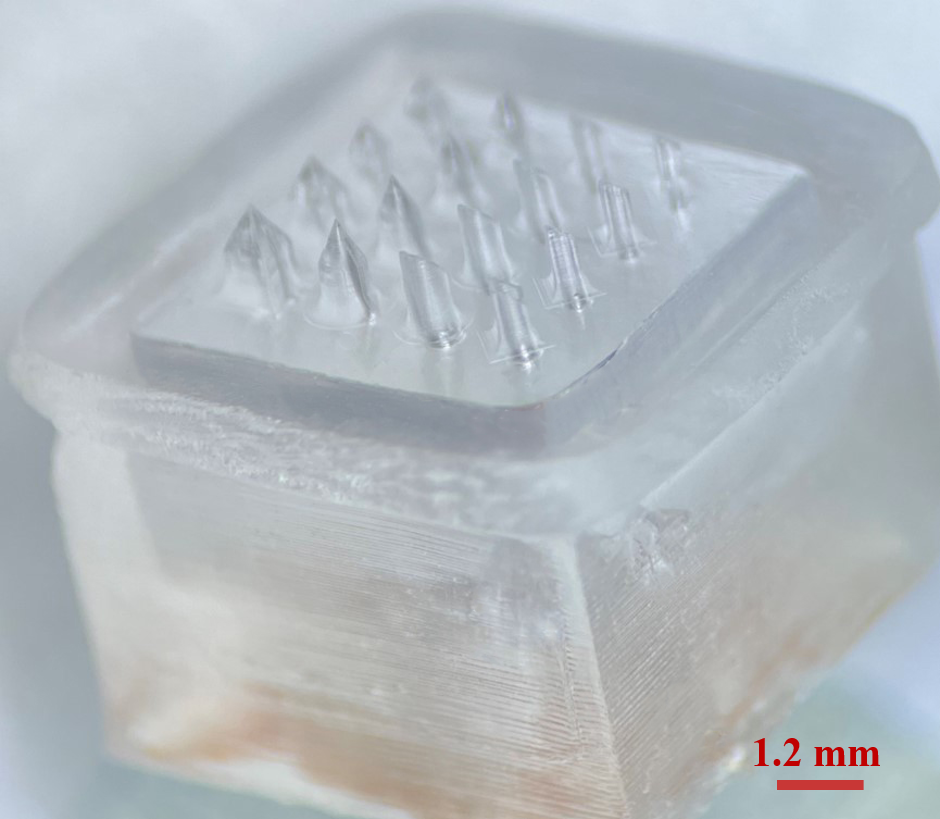


**Fig. S1.** Close-up view of the tip of light-curing resin HMNs.


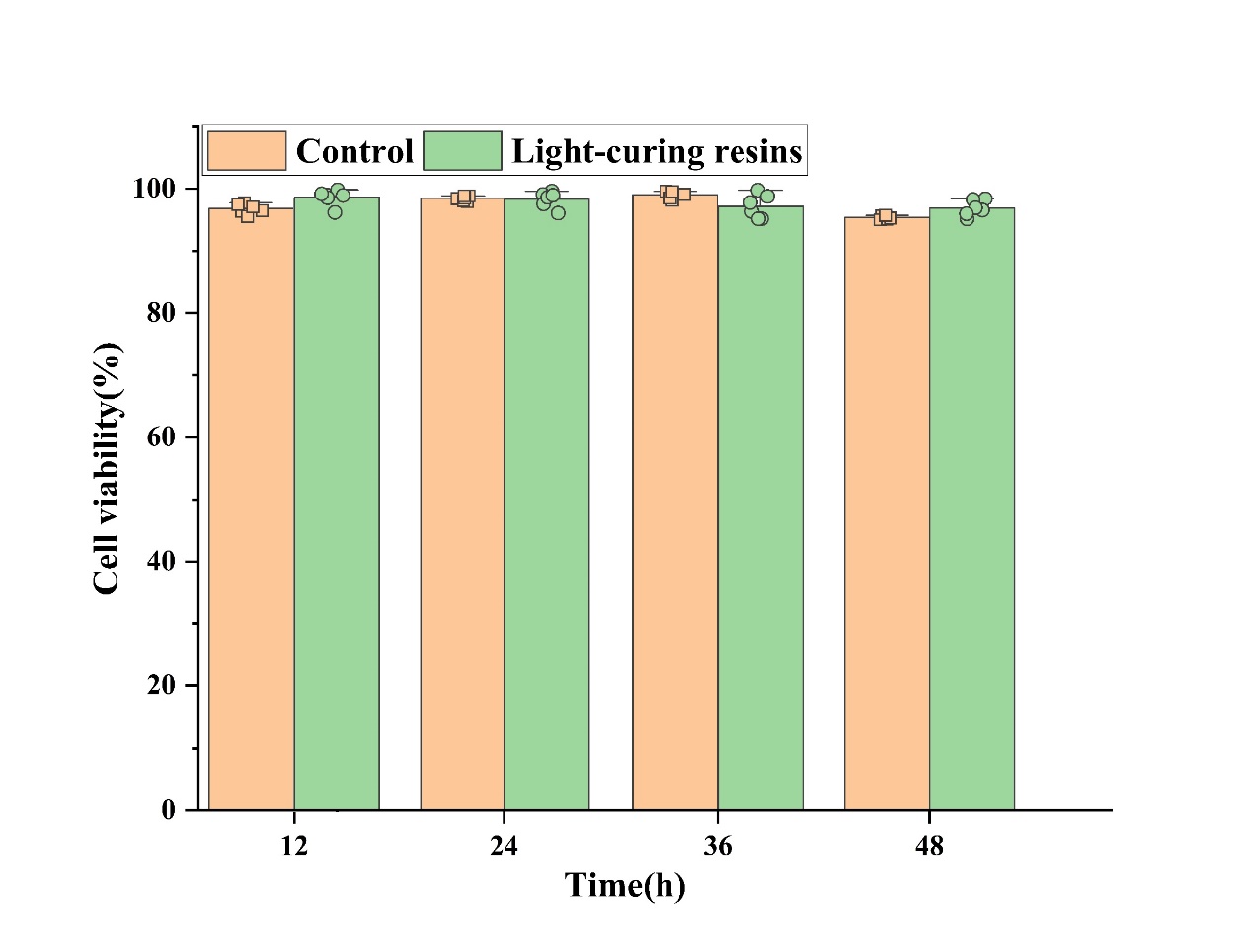


Fig. S2. Cytotoxicity verification of the resin-HMNs showed that the cellular activity was consistently maintained at different time points similar to the control, verifying the biocompatibility of the material.


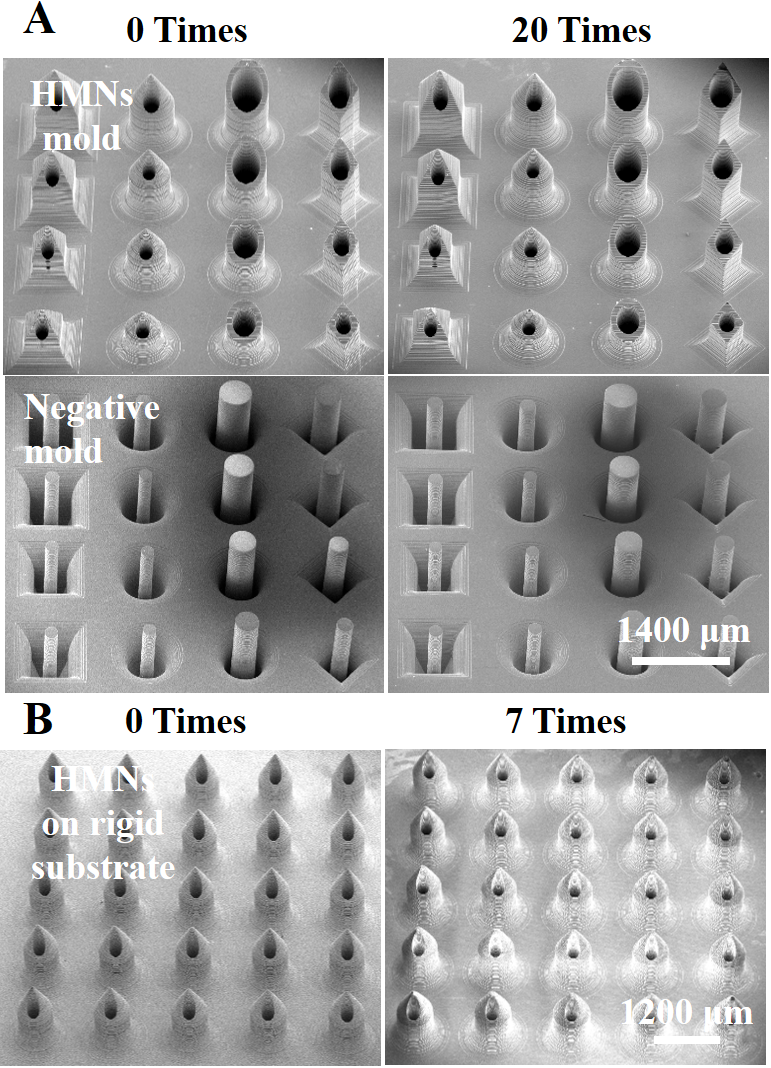


Fig. S3. (A) The 3D printed master mold and negative mold showed almost no change in shape after 20 uses. (B) The HMNs maintained their original shape after 7 uses.


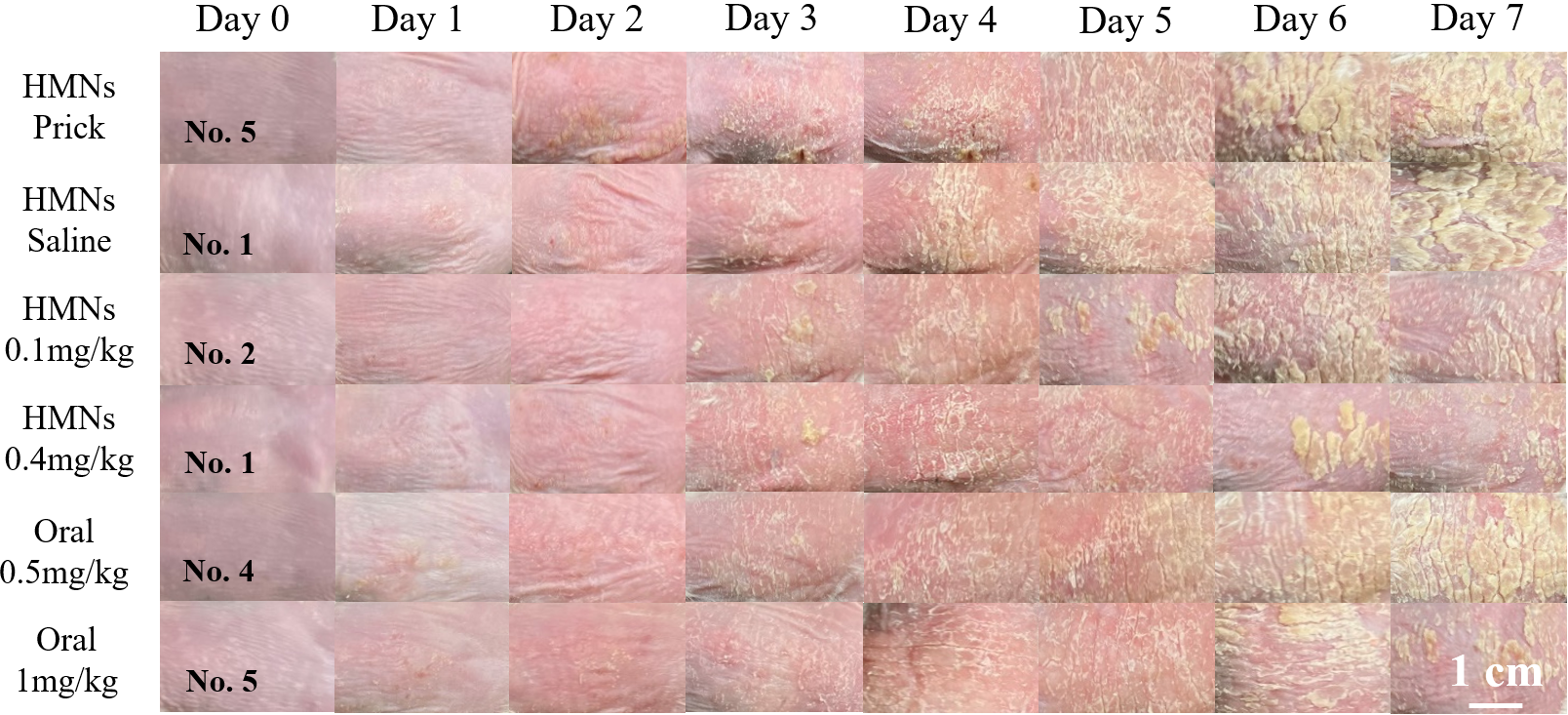


Fig. S4. Visualization of the left dorsal skin of mice.

| **PASI Score** | | | | | | | | |
| --- | --- | --- | --- | --- | --- | --- | --- | --- |
| **Days**  **Groups** | 0 | 1 | 2 | 3 | 4 | 5 | 6 | 7 |
| Normal | 0 | 0 | 0 | 0 | 0 | 0 | 0 | 0 |
| Model | 0 | 0 | 1 | 2 | 3 | 4 | 4 | 4 |
| HMNs+Prick | 0 | 0 | 1 | 2 | 3 | 4 | 4 | 4 |
| HMNs+Saline | 0 | 0 | 1 | 2 | 3 | 4 | 4 | 4 |
| HMNs+0.1 mg/kg | 0 | 0 | 1 | 2 | 3 | 3 | 4 | 3 |
| HMNs+0.2 mg/kg | 0 | 0 | 1 | 1 | 2 | 2 | 1 | 0 |
| HMNs+0.4 mg/kg | 0 | 0 | 1 | 2 | 2 | 3 | 2 | 1 |
| Oral+0.5 mg/kg | 0 | 0 | 1 | 2 | 2 | 3 | 4 | 4 |
| Oral+1 mg/kg | 0 | 0 | 1 | 1 | 2 | 2 | 3 | 2 |
| Oral+2 mg/kg | 0 | 0 | 1 | 1 | 2 | 2 | 1 | 0 |

Table S1. PASI was assessed by unrelated personnel based on the appearance of the skin.


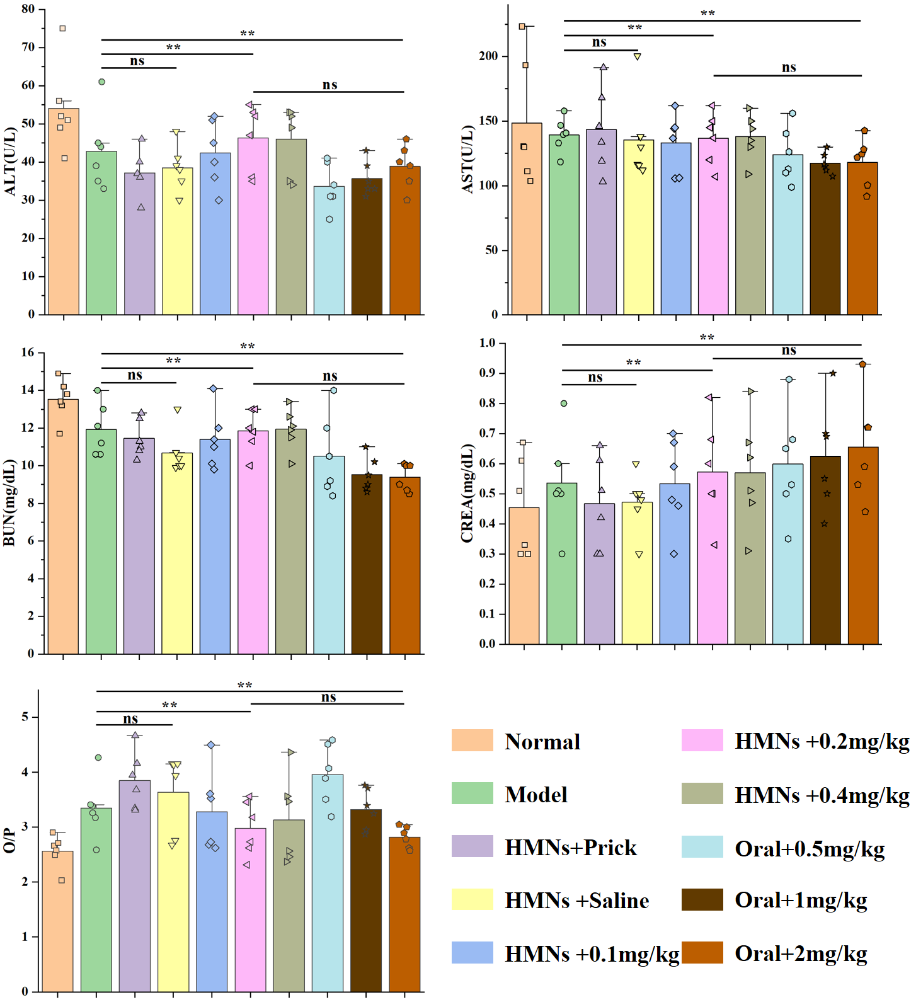


Fig. S5. Serum index analysis: ALT and AST reflect liver function. BUN and CREA reflect renal function. O/P reflects the degree of health. *(n*=6, mean ± *SDs*) (*ns*, “*”, “**”, and “***” indicate no significance, *P*<0.05, *P*<0.01, and *P*<0.001.)


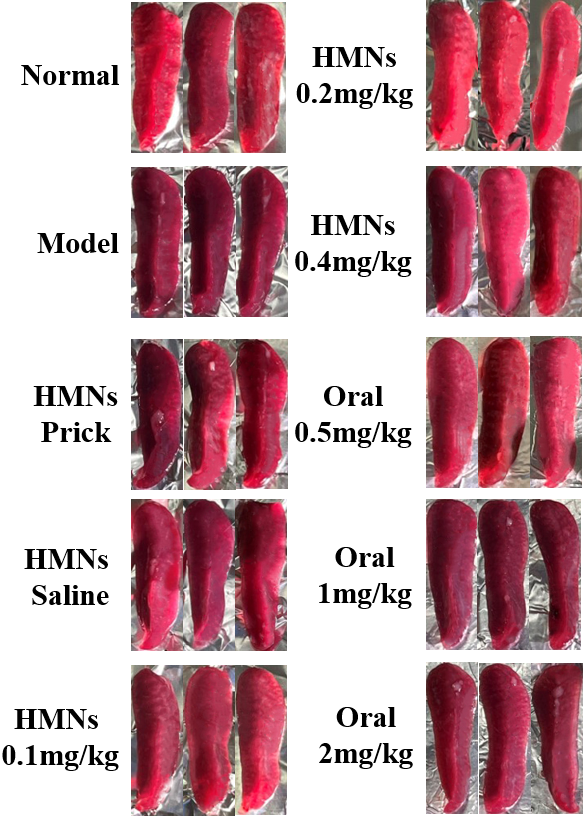


Fig. S6. Kidney sampling in different groups.
